# Experimental Studies of the Molecular Pathways Regulated by Exercise and Resveratrol in Heart, Skeletal Muscle and the Vasculature

**DOI:** 10.3390/molecules190914919

**Published:** 2014-09-18

**Authors:** Vernon W. Dolinsky, Jason R. B. Dyck

**Affiliations:** 1 Department of Pharmacology & Therapeutics and the Diabetes Research Envisioned and Accomplished in Manitoba (DREAM) Research Theme of the Manitoba Institute of Child Health, University of Manitoba, 601 John Buhler Research Centre, 715 McDermot Avenue, Winnipeg, MB R3E 3P4, Canada; 2 Department of Pediatrics and the Cardiovascular Research Centre, Mazankowski Alberta Heart Institute, University of Alberta, 458 Heritage Medical Research Centre, Edmonton, AB T6G 2S2, Canada

**Keywords:** resveratrol, exercise, mitochondria, reactive oxygen species, nitric oxide

## Abstract

Regular exercise contributes to healthy aging and the prevention of chronic disease. Recent research has focused on the development of molecules, such as resveratrol, that activate similar metabolic and stress response pathways as exercise training. In this review, we describe the effects of exercise training and resveratrol on some of the organs and tissues that act in concert to transport oxygen throughout the body. In particular, we focus on animal studies that investigate the molecular signaling pathways induced by these interventions. We also compare and contrast the effects of exercise and resveratrol in diseased states.

## 1. Introduction

Once O_2_ enters the body via the lungs, its transport throughout the body is performed by the heart, blood and vasculature. These organ systems form an integrated unit that is responsible for the transport and utilization of O_2_ for the eventual synthesis of ATP by cells [1]. The O_2_ transport unit of the body can be maintained and/or improved by exercise [2,3] and there is clear evidence that moderate exercise and an active lifestyle are useful in the prevention of several chronic diseases including metabolic syndrome [4], type 2 diabetes [5], cardiovascular disease [4], and neurodegenerative disorders [6]. While exercise training functionally optimizes the O_2_ transport unit, various nutritional, hormonal or small molecule treatments are being investigated to mimic the beneficial effects of exercise on the organ systems that comprise the O_2_ transport unit. One of these natural molecules is resveratrol.

Resveratrol is a polyphenol produced by plants in response to environmental stress [7]. Although present in small amounts in many plant-based foods, nutritional supplements of resveratrol are often produced from extraction of resveratrol from the dried roots of *Polygonum cuspidatum* (knotweed), which has also been used in traditional Asian medicine to treat cardiovascular disorders [8]. The *trans*-isomer of resveratrol is an effective anti-oxidant that scavenges free radicals [9]. Pharmacological studies suggest that therapeutic doses of resveratrol are non-toxic and well tolerated by humans, though mild to moderate gastrointestinal adverse effects such as diarrhea, nausea, and abdominal pain were reported in individuals administered greater than one gram of resveratrol [10]. One important caveat is that to date, clinical trials of resveratrol have been short-term (mostly a few months in length), so the safety of long-term resveratrol consumption is unknown. Resveratrol is rapidly and efficiently absorbed following oral administration, though its bioavailability is low due to its metabolism to sulfated and glucoronidated derivates during first pass metabolism by the liver [11,12]. Despite this rapid clearance, studies in animals and in humans have shown that oral administration of resveratrol protected against the development of various cardiovascular and metabolic diseases [13,14,15,16,17,18].

While resveratrol is widely considered to be a molecule that acts as a calorie restriction mimetic (reviewed in [13]), resveratrol also causes adaptations in the O_2_ transport system that are similar to exercise training. In many cases, resveratrol activates molecular pathways that are analogous to the effects of exercise training [19,20,21]. However, resveratrol has also been shown to augment the effects of exercise training [22,23], suggesting that resveratrol affects additional molecular pathways that are not affected by exercise training. Conversely, recent human studies have shown that resveratrol may prevent the beneficial effects of exercise in certain patient populations [24,25], thus not only questioning the effectiveness of resveratrol as an exercise mimetic but also suggesting that it may have detrimental effects. 

This review will compare the effects of exercise training and resveratrol on the coordinative adaptation of the organs that comprise the O_2_ transport system and the molecular pathways that are involved. In addition, we will review the recent literature that has directly compared the effects of exercise and resveratrol treatments in disease and also examined the effects of combining resveratrol with exercise training.

## 2. Resveratrol and Exercise in Rodent Models

An active lifestyle is considered to be an important component of healthy aging [26]. Since resveratrol has been suggested to be an exercise mimetic [19,20,21], it stands to reason that resveratrol may also play a role in improving health during aging and/or extending lifespan. In agreement with this, resveratrol has been shown to increase exercise endurance in a mouse model of accelerated aging [27] and can increase lifespan in rodents [28,29]. While resveratrol likely has numerous beneficial effects in these settings (reviewed in [30]), it is clear that skeletal muscle function can be significantly enhanced by resveratrol treatment. For example, administration of resveratrol to sedentary high fat fed mice increased skeletal muscle endurance and strength in these mice [28,29]. Moreover, resveratrol prevented muscle wasting resulting from mechanical unloading [31], improved the recovery of muscle mass following disuse in rats [32] and improved muscle function/reduced muscle damage in the mdx mouse model of Duchenne muscular dystrophy [33]. Together, these findings demonstrate that resveratrol has beneficial effects in rodents that have an impaired ability to perform exercise.

Interestingly, not only does resveratrol improve exercise capacity in rodents with underlying chronic health conditions, but resveratrol also enhanced skeletal muscle endurance and strength in healthy young adult rats [22] and mice [34]. Similarly, resveratrol improved aerobic endurance and muscle strength in rats that were selectively bred for high capacity running [23]. The mechanisms responsible for the effects of resveratrol on strength and endurance parallel the adaptations observed in response to exercise training, suggesting that common pathways are activated by exercise as well as resveratrol. However, resveratrol and exercise training may also regulate separate cellular pathways since we have recently demonstrated in two separate rodent models that resveratrol has effects on the heart that exceed those observed with exercise training alone [35]. In addition, resveratrol has additive effects on the heart and skeletal muscle when administered in combination with exercise training [22]. In contrast to the preceding studies, resveratrol is not beneficial in all rodent models tested. For example, resveratrol did not improve endurance in genetically obese mice that were fed resveratrol and subjected to a single bout of exercise [36]. More recently, exercise training and resveratrol were directly compared in rats that were selectively bred for low capacity running and it was shown that resveratrol did not improve treadmill endurance even though 12-weeks of exercise training did improve endurance [37]. Together, these findings suggest that our understanding about the effects resveratrol as an exercise mimetic has not been clearly defined.

## 3. Resveratrol, Exercise and Mitochondrial Adaptations

Muscle tissues require large amounts of energy (in the form of ATP) for contractile function during physical activity. A sizeable quantity of ATP can be generated by muscle mitochondria via oxidative phosphorylation. Consequently, heart and skeletal muscle mitochondrial content and function define both athletic performance and cardiovascular health. Indeed, one of the beneficial adaptations by muscle to exercise training is increased mitochondrial function and content [38,39,40]. Similar to exercise, resveratrol also promotes mitochondrial biogenesis in endothelial cells [41], skeletal muscle and cardiac tissues [42,43]. However, aging as well as metabolic and cardiovascular disease [44,45,46] reduce mitochondrial function and/or content in muscle tissue, thereby reducing exercise capacity. Importantly, both exercise training and resveratrol can increase mitochondrial function and content in aging and thus prevent the aging-associated decline in skeletal muscle mitochondria content [47,48].

Multiple mechanisms may explain exercise-induced mitochondrial biogenesis. One factor that is likely involved is the master-regulator of mitochondrial biogenesis, peroxisome proliferator activated receptor (PPAR) gamma coactivator (PGC)1α. Indeed, increased nuclear abundance of PGC1α is observed in exercise trained human skeletal muscle [49,50] signifying increased oxidative metabolism, type I muscle fibers and transcriptional expression of mitochondrial proteins. Like exercise, the resveratrol-mediated effects on mitochondrial biogenesis also appear to involve PGC1α-regulated transcription. For instance, resveratrol has been reported to activate PGC1α through its ability to activate pathways that are sensitive to the energy status of the cell. One of these pathways is the NAD-dependent protein deactylase, SIRT1 [28,51], which deacetylates and stimulates PGC1α in skeletal muscle. The dependence of resveratrol on SIRT1 to stimulate PGC1α in skeletal muscle was demonstrated in SIRT1 deficient mice. In this model, SIRT1 was necessary for the stimulation of mitochondrial biogenesis and PGC1α nuclear translocation by voluntary exercise as well as resveratrol [52]. However, the role of SIRT1 in PGC1α stimulation was recently brought into question since the inhibition of SIRT1 in C2C12 myotubes increased PGC1α acetylation and also increased expression of mitochondrial proteins [53]. In this same study, SIRT1 overexpression in the C2C12 myotubes and rat triceps muscle reduced PGC1α acetylation and mitochondrial protein expression [53]. Together, although these latter findings suggest that SIRT1 may not be involved in PGC1α stimulation, other studies demonstrate that resveratrol and exercise training do act through similar PGC1α-dependent mechanisms to stimulate mitochondrial biogenesis (Table 1).

**Table 1:** Effects of exercise and resveratrol on molecular pathways.

	Exercise Training				Resveratrol
Mitochondrial content/function					
⇑	PGC1α	49, 50, 64	⇑	PGC1α	15, 28, 51, 64
⇑	SIRT1	28	⇑	SIRT1	15, 28, 51, 52
⇑	AMPK	57, 58, 61, 62, 63	⇑	AMPK	22, 23
Reduced blood pressure					
⇑	eNOS	96, 97	⇑	eNOS	80, 81, 91, 92, 93, 94, 95
⇓	ET-1	98	⇓	ET-1	91, 99, 100, 101
⇓	Ang-II	98	⇓	Ang-II	101
?	SIRT1		⇑	SIRT1	41, 104, 105
⇑	AMPK	106	⇑	AMPK	77, 95
?	LKB1		⇑	LKB1	95
⇑	SOD2	109, 110, 111	⇑	SOD2	112, 113, 114
⇓	NFkB	120	⇓	NFkB	80, 121
⇓	COX-2	128	⇓	COX-2	127
Left-ventricular hypertrophy					
⇑	p70 S6 kinase	140	⇓	p70 S6 kinase	148
⇑	eEF2	140	⇓	eEF2	148
⇑	AMPK	61, 62	⇑	AMPK	95, 117, 135, 148
?	LKB1		⇑	LKB1	95, 117
⇔	NFAT	143	⇓	NFAT	148
Ischemia-Reperfusion Injury					
⇑	Akt	159, 160	⇑	Akt	76
⇑	SOD2	162	⇑	SOD2	76, 169, 172
⇑	eNOS	161	⇑	eNOS	167, 168
Heart Failure					
⇔	SIRT1	35	⇑	SIRT1	177, 179, 187
⇑	SOD2	162	⇑	SOD2	35, 177, 187
?	PGC1α		⇑	PGC1α	42
⇔	AMPK	35	⇑	AMPK	187, 195

**Figure 1:**
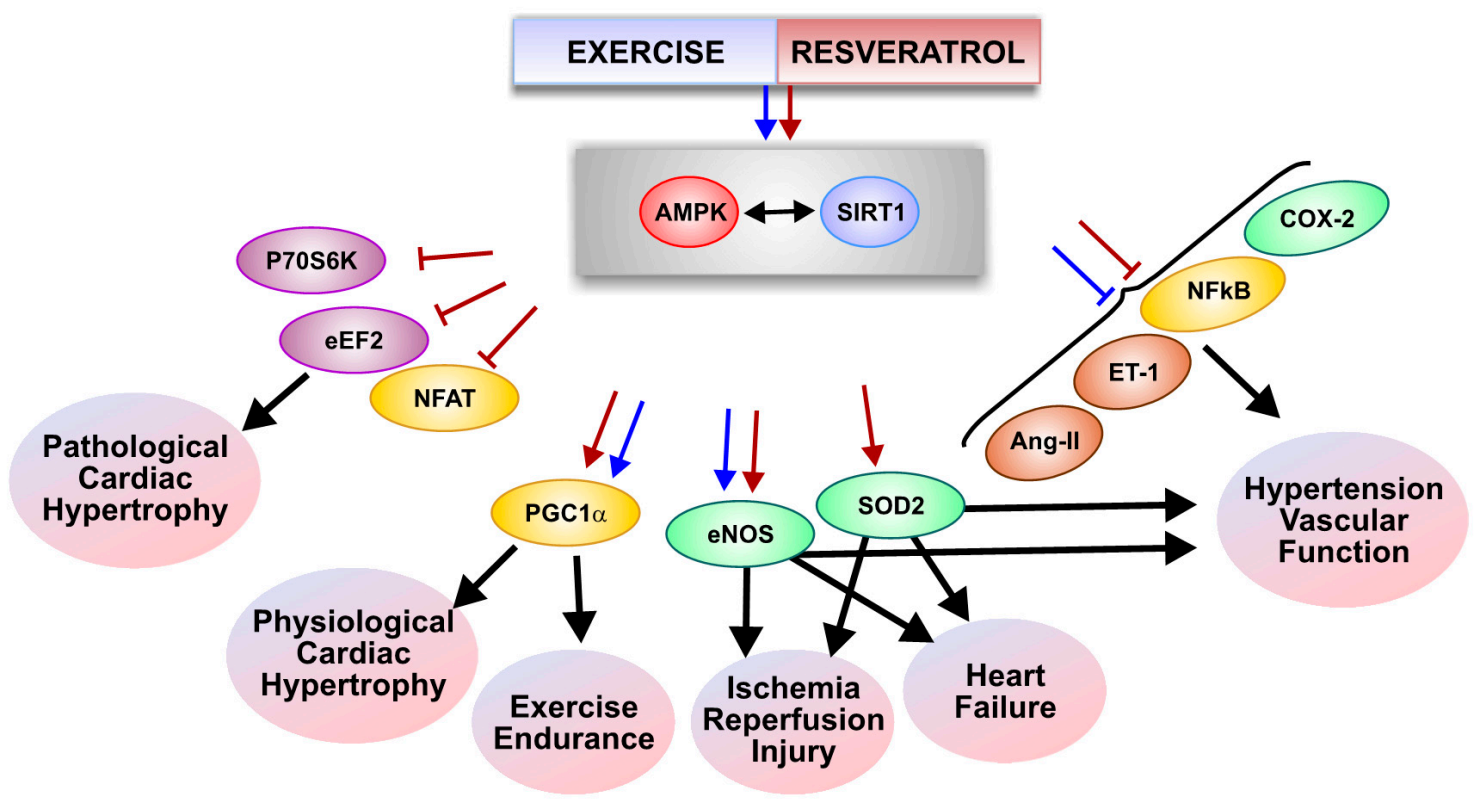
Molecular pathways regulated by exercise training and resveratrol in the heart, skeletal muscle and vasculature. Blue arrows/lines indicate molecular pathways activated by exercise and red arrows/lines indicate molecular pathways activated by resveratrol. AMPK, AMP-activated protein kinase; Ang-II, angiotensin-II; COX-2, cyclooxygenase-2; eEF2, eukaryotic elongation factor2; eNOS, endothelial nitric oxide synthase; ET-1, endothelin-1; NFAT, nuclear factor of activated T-cells; NFkB, nuclear factor kB; p70S6K, p70 S6 kinase; PGC1α, peroxisome proliferator activated receptor gamma coactivator; SIRT1, sirtuin-1; SOD2, superoxide dismutase-2.

In addition to the potential of a direct SIRT1-PGC1α signaling pathway as a mediator of the beneficial effects of exercise in skeletal muscle, it appears that activation of the AMP-activated protein kinase (AMPK) is also involved in regulating the adaptations by muscle tissues to exercise [54,55,56,57,58]. AMPK is an energy sensing kinase that controls a variety of metabolic cellular processes [59,60]. Evidence for AMPK being involved in skeletal muscle adaptations to exercise was obtained in mice with a skeletal muscle tissue specific knock-out of the AMPKα subunits [57]. These mice have an impaired exercise capacity and significantly reduced muscle tissue mitochondrial respiration [57]. A similar impairment in exercise-induced adaptations was observed when the AMPKβ subunits were deleted in skeletal muscle thus creating a profound AMPK deficiency [58]. As exercise stimulates AMPK activity in muscle [61,62,63], this pathway may also be stimulated by resveratrol. Indeed, resveratrol stimulated skeletal muscle AMPK phosphorylation and increased exercise endurance in normal Wistar rats [22] and rats selectively bred for high capacity running [23]. In addition, administration of resveratrol at a dose where its bioavailability is below the level required to activate AMPK failed to stimulate mitochondrial biogenesis [53], suggesting the need for AMPK activation for resveratrol-induced mitochondrial biogenesis. Consistent with this, resveratrol failed to stimulate AMPK and improve exercise endurance in rats selectively bred for low capacity running [37]. Whether or not AMPK signals through additional pathways or through the activation of PGC1α in muscle tissues has not been clearly established. However, the failure of resveratrol to activate AMPK in certain models could explain the inability of resveratrol to improve endurance in these studies. Notwithstanding this, many of the effects of exercise and resveratrol in skeletal muscle were determined to be PGC1α-dependent [64], suggesting that the effector molecule responsible for improved mitochondrial function with resveratrol treatment is PGC1α, regardless of how it is activated (*i.e.*, SIRT1, AMPK or both, Figure 1). That said, numerous other mechanisms could also contribute to resveratrol-mediated improvements in muscle function including mitochondrial fusion, which has a beneficial role in muscle endurance [45]. Mitochondrial fusion enables mitochondria to mix their contents within an interconnected mitochondrial reticulum and optimize function. In skeletal muscle [37] and the heart [35,42], both exercise training and resveratrol have been demonstrated to increase the expression mitofusin-1 and mitofusin-2 that act to regulate the process of mitochondrial fusion and fission. As such, the precise molecular mechanisms involved in resveratrol/exercise mediating all of these effects have not yet been clearly defined.

## 4. Resveratrol and Exercise in Human Clinical Trials

The research described above suggests that resveratrol has similar effects as exercise training and in some rodent models the effects of resveratrol may enhance or even exceed the effects of moderate exercise training. However, a recent small clinical trial recruited physically inactive but otherwise healthy men (aged 60–75) and investigated the effects of resveratrol or placebo in combination with high-intensity aerobic exercise training [24,25]. Following exercise training muscle endurance and maximal oxygen uptake were increased while skeletal muscle oxidative stress and inflammation were reduced in the placebo group [24,25]. The exercise-induced improvements in these parameters were largely absent in the resveratrol group [24,25]. While the focus of the trial was on exercise, skeletal muscle and circulating factors, cardiac and vascular imaging were not performed to assess the effects of resveratrol during exercise training on these systems [24,25]. As such, it is difficult to predict why this trial was negative. However, it is possible that dosages may account for these differences as the ~3.1 mg/kg/day used in this trial [24,25] is significantly less the >100 mg/kg/day used in the rodent studies [22,23,27,35,52], suggesting that the dosage used in the Gliemann *et al.* and Olesen *et al.* [24,25] clinical trial could be below the level required to activate the molecular targets described in Section 3. That said, a lower dosage of resveratrol (~1.5 mg/kg/day) induced positive skeletal muscle mitochondrial adaptations and activated PGC1α in overweight sedentary men [15]. Given that the men recruited in the Gliemann *et al.* and Olesen *et al.* trial [24,25] did not have preexisting health conditions, it is also possible that poor cardiometabolic health is a prerequisite for observing resveratrol induced improvements with exercise training in humans. Alternatively, the high intensity nature of the aerobic exercise performed by the participants [24,25] may also have influenced the effects of resveratrol in the aged population examined. While regular and moderate physical exercise is clearly beneficial, long-lasting high intensity exercise can have harmful effects [65,66,67,68]. For example, bouts of exercise that are too heavy or are not followed by sufficient rest periods can induce oxidative stress, inflammation and muscle damage [69]. During vigorous exercise, intracellular substrate stores are used for oxidation. The combination of resveratrol and high-intensity aerobic training may deplete these stores in the aged and consequently sufficient levels of substrates are not available to observe any potential additive effects of resveratrol on exercise capacity. As such, it is possible that the addition of resveratrol to high intensity exercise training may have had the same effect as vigorous exercise that exceeds the threshold where it is adaptive, thereby blunting the positive effects of exercise on the measured parameters. While the results from the aforementioned trials are clear, more human trials are needed to study the effects of resveratrol on exercise training and cardiovascular health to answer some of these questions.

## 5. Resveratrol Regulates Vascular Function and Attenuates Hypertension

Exercise training improves the vasodilatory properties of the vasculature thereby optimizing O_2_ transport throughout the body [70,71]. Several studies have shown that the acute intake of polyphenolic compounds in red wine or grape extracts also increases flow-mediated vasodilation in patients with pre-existing cardiovascular disease [72,73]. Given the high levels of resveratrol in these extracts, the effects of purified resveratrol were initially examined in endothelial cell cultures and resveratrol was demonstrated to increase the production of vasodilatory molecules and reduce the production of pro-inflammatory molecules [74,75]. Specifically, treatment of isolated carotid arteries [76], as well as aortic rings [77] with resveratrol stimulated endothelial-dependent vasodilation. In addition, the chronic treatment of normotensive as well as hypertensive rats with resveratrol increased the vasodilation and compliance of isolated aortic rings [78,79,80,81] and mesenteric arteries [82,83]. Resveratrol also prevented vascular injury caused by revascularization in rats and mice through the inhibition of neointimal formation [84]. Recently, these effects of resveratrol have also been observed in humans where resveratrol induced both endothelium-dependent as well as endothelium-independent vasodilation in vessels isolated from non-hypertensive patients [85,86]. In addition, resveratrol improved flow-mediated vasodilation in overweight individuals [17,87], as well as in mildly hypertensive and overweight adults [88]. However, the beneficial effects of resveratrol on vascular function in humans appeared to be dependent upon a pre-existing metabolic health condition, as resveratrol did not affect endothelial function or vascular stiffness in healthy middle-aged non-obese individuals [89].

Although all of the effects of resveratrol on the vasculature remain to be characterized, in some studies the vasodilatory effects of resveratrol have been attributed to its inherent anti-oxidant properties [78,90]. In addition, the vasodilatory effects of resveratrol involve increased bioavailability of nitric oxide (NO). Indeed, it has been shown in endothelial cell cultures [80,81,91,92,93,94] and vessels isolated from rodents [95] that increased expression/activity of endothelial NO synthase (eNOS) in response to resveratrol treatment is a critical factor in increasing NO levels. These effects are similar to exercise training (Table 1), where resveratrol-induced increase of NO bioavailability involved higher expression/activity of eNOS in hypertensive rats [96] and humans [97].

In addition to its effects on NO, the vasodilatory effects of exercise training also involve reduced levels of molecules that cause vasoconstriction [98]. In both cultured cells and rodent models, resveratrol reduces the expression of endothelin-I [91,99,100,101], angiotensin-II [101] and the angiotensin-II type 1 receptor [102]. For example, resveratrol treatment of isolated aorta preparations blocked angiotensin-II induced contractions [103]. Moreover, resveratrol treatment of mice infused with angiotensin-II markedly improved flow-mediated vasodilation of the femoral artery* in vivo* [95]. Taken together, these findings suggest that, similar to exercise, resveratrol mediates positive adaptations to vascular function.

The mechanism for increased NO production by resveratrol in endothelial cells has been proposed to involve SIRT1. SIRT1 deacetylates eNOS at lysine residues, thereby stimulating NO production [104,105]. Knock-down of endogenous SIRT1 levels using RNAi attenuated resveratrol-induced increases in eNOS mRNA and protein expression [41]. However, AMPK also appears to be involved in this process as acute exercise activates eNOS as well as AMPK in the mouse aorta [106] and AMPK is able to directly phosphorylate eNOS on serine 1177, which increases NO production [107,108]. In addition, resveratrol activated AMPK and pharmacological inhibition of AMPK abolished the ability of resveratrol to improve endothelium dependent vasodilation in aortic rings [77]. Consistent with the findings in rodents, stimulation of NO production and eNOS by resveratrol was demonstrated to require the activation of AMPK in superior thyroid arteries isolated from hypertensive patients [85]. These results provide compelling evidence of the importance of AMPK in the resveratrol-mediated effects on vascular relaxation. 

Regular exercise training also improves vascular function in association with the reduction of reactive oxygen species [69]. Exercise training increases the expression of antioxidant enzymes such as superoxide dismutase (SOD), catalase and glutathione peroxidase [109,110,111]. Similar to exercise, resveratrol increases the expression of these antioxidant enzymes in endothelial [112,113] and smooth muscle cells [114]. Recently, resveratrol has also been shown to reduce vascular damage via increased expression of SOD2 in vessels isolated from hypertensive patients [85]. This property of resveratrol appeared to be dependent upon the activation of the nuclear factor erythroid-derived 2-like 2 transcription factor (Nrf2) [85]. Unlike exercise, resveratrol also scavenges reactive oxygen species [78,113], suggesting that resveratrol provides additional benefit for alleviating oxidative stress beyond exercise training. Resveratrol also dose-dependently increases SIRT1 levels, which could be of significant benefit over exercise as SIRT1 overexpression has been shown to attenuate mitochondrial oxidative damage in endothelial cells [115,116]. However, it is currently unknown whether SIRT1 levels are also increased in the arteries of exercise-trained mice in association with reduced reactive oxygen species. Lastly, the inhibition of the kinase upstream of AMPK, termed liver kinase B (LKB)1, by the lipid peroxide, 4-hydroxyl-2-nonenal (HNE) [117,118] could provide a connection between excess reactive oxygen species and the inhibition of an LKB1-AMPK-eNOS signaling cascade that causes reduced NO bioavailability and impaired vasodilatory responses in hypertension [95]. Indeed, resveratrol treatment of hypertensive rodents reduced systemic levels of HNE, rescued arterial LKB1-AMPK-eNOS activity and attenuated high blood pressure [95], suggesting that resveratrol can improve reactive oxygen species-mediated impaired AMPK-eNOS signaling.

The nuclear factor kappa B (NFkB) is a key transcription factor that is activated by reactive oxygen species and stimulates the expression of inflammatory cytokines. Therefore, activation of NFkB connects oxidative stress with systemic inflammation and could be a significant factor that contributes to the development of vascular disease. The anti-inflammatory properties of regular exercise are well documented [119] and may involve the inhibition of the NFkB-mediated transcription of pro-inflammatory cytokines [120]. Like exercise, resveratrol inhibits NFkB activation [80,121] and consequently contributes to the reduced expression of interleukins (IL)-6 and IL-8 [80,122,123,124,125] and inhibition of tumour necrosis factor-α [80,126]. Resveratrol and exercise also inhibited cyclooxygenase (COX)2 expression [127,128] and reduced the production of inflammatory molecules such as prostaglandin E_2_ [127,128]. Consistent with these effects observed in rodents, it was recently demonstrated that dietary resveratrol prevented high fat and sucrose diet-induced vascular stiffening, oxidative stress and inflammation in rhesus monkeys [129]. Together, these findings suggest that the anti-inflammatory effects of both regular exercise and resveratrol are likely important factors in their positive effects on the vascular system (Table 1).

Vascular dysfunction is associated with high systemic blood pressure. As mentioned, resveratrol has been shown to increase NO concentrations as well as reduce oxidative stress, inflammation and vasoconstriction molecules, which together protect against increased blood pressure. However, the effects of resveratrol on blood pressure are controversial with some studies reporting that resveratrol reduces blood pressure and others report that resveratrol has no effect. For example, resveratrol administration reduces blood pressure in cholesterol-fed swine [130], obese Zucker rats [126], fructose-fed rats [93], the DOCA-salt sensitive rat [125,131], the partially nephrectomized rat [101], two-kidney one-clip hypertensive rat [81], angiotensin-II infused mice [95,103], transgenic rats that overexpress the human renin and angiotensinogen genes [43], as well as in a rat model of pulmonary hypertension [132]. On the other hand, 2.5 mg/kg of resveratrol did not affect the blood pressure of the SHR [79,117,133], stroke prone-SHR rats [134] or the Dahl-salt sensitive rat [42], though a longer duration of a higher dose of resveratrol attenuated hypertension in the SHRs [82,95]. However, the combination of low dose resveratrol with hydralazine enhanced the blood pressure lowering efficacy of hydralazine in 28 week-old SHRs with established hypertension [135], suggesting that there are vascular effects of resveratrol even at lower doses. Overall, these findings suggest that the differences in the* in vivo* effects of resveratrol in animal models of hypertension are likely a consequence of different mechanisms for increasing blood pressure in the various animal models, the dose of resveratrol administered and/or the length of treatment.

Similar to the findings in rodents, clinical trials of resveratrol have indicated that there is a dose-dependent effect on blood pressure in humans. A minimum dose of 150 mg resveratrol reduced systolic blood pressure in non-hypertensive subjects [14,15,82], while lower doses had no effect on blood pressure in humans [87,136,137]. Given the positive effects of resveratrol on vascular function in humans and its generally beneficial effects in different hypertensive rodent models, the effects of resveratrol on blood pressure should be examined in hypertensive populations.

## 6. Resveratrol and Exercise in Physiological Cardiac Hypertrophy

Left ventricular hypertrophy (LVH) predominantly develops in response to increased cardiac workload and may be broadly divided into physiological or pathological hypertrophy (see Section 7 for the latter). Physiological LVH is an adaptive response of the myocardium to physiological stresses, including exercise training [138]. Physiological LVH involves growth of the myocardium in response to increased workload and thus considerable overlap exists (Figure 1) between the mechanisms that control the pathological growth of the heart and the growth of the heart in response to exercise training [138]. For example, increased cardiomyocyte cell size and protein synthesis are properties of both physiological and pathological LVH [139]. Similar to pathological LVH, exercise training increases p70S6K and eEF2 phosphorylation in the heart [140]. In the case of physiological LVH, this growth does not result in impaired myocardial contractility [141] or other deleterious consequences that are associated with pathological LVH [142]. Unlike pathological LVH, physiological LVH does not involve the calcineurin-nuclear factor of activated T-cells (NFAT) pathway [143] and training increases myocardial AMPK activity [61,62]. Since LVH is a positive adaptation to exercise training, it appeared plausible that resveratrol treatment during exercise could block the physiologic growth of the myocardium and be deleterious. Until recently, little was known regarding the effects of resveratrol on the physiological growth of the heart. Interestingly, resveratrol did not affect the growth of the myocardium in rats subjected to 12-weeks of 60-min of daily aerobic treadmill exercise [22] or in a rat model of volume overload-induced [144] cardiac hypertrophy. While the mechanisms that define the distinct effects of resveratrol on the myocardium in pathological and physiological situations remain to be defined, it is likely that the stimuli that promote pathological LVH work through different signaling pathways than those that promote physiological LVH and that resveratrol acts to primarily inhibit those pathways that promote pathological LVH (Table 1).

## 7. Resveratrol and Exercise in Pathological Cardiac Hypertrophy

Given the ability of resveratrol to modulate vascular function and prevent the development of high blood pressure in some rodent models of hypertension, the effect of resveratrol on the development of pathological cardiac hypertrophy was examined. As expected, resveratrol prevented LVH via reduced blood pressure in two-kidney one-clip [81] and partially nephrectomised rats [101]. In addition, resveratrol prevented agonist-induced LVH in DOCA-salt treated rats [131], rats that overexpress the human renin and angiotensinogen genes [43] and in angiotensin-II infused mice [95], which also appeared to involve reduced blood pressure.

While increased hemodynamic load is the major factor in the development of pathological LVH [141], alterations in molecular signalling pathways (Figure 1) may also contribute to increased cardiomyocyte growth in conjunction with and/or in the absence of increased afterload [145]. At low doses, resveratrol prevented the development of LVH without affecting blood pressure in the SHR rat [117,133], in surgical models of pressure overload due to abdominal aortic banding [144,146] and transverse aortic constriction [147]. Therefore, resveratrol appears to also have direct effects on the growth of the cardiomyocyte independent from reducing hemodynamic load. Indeed, experiments utilizing isolated cardiomyocytes have demonstrated that resveratrol inhibits phenylephrine [148] and norepinephrine [135] induced cellular hypertrophy and these effects involve the inhibition of protein synthesis. 

The proteins p70 S6 kinase (p70S6K) and eukaryotic elongation factor-2 (eEF2) regulate protein synthesis via translation. Resveratrol has been shown to act to inhibit p70S6K and eEF2 activity and consequently protein synthesis via the activation of AMPK as well as the inhibition of Akt [148]. Whether AMPK activation or Akt inhibition was necessary for the effects of resveratrol was examined in mouse embryonic fibroblasts (MEFs) deficient in either AMPK or Akt. While the anti-growth effects of resveratrol were largely preserved in Akt null MEFs, resveratrol was significantly less effective in AMPK null MEFs [148]. In addition, an AMPK inhibitor blocked the ability of resveratrol to reduce cardiomyocyte size and protein synthesis in adult rat cardiomyocytes [135]. Moreover, the inhibition of p70S6K and the prevention LVH by resveratrol in SHRs and angiotensin-II infused mice involved the activation of AMPK in the absence of Akt inhibition [95,117,135]. In addition, high levels of the lipid peroxidation by-product, HNE, in hypertrophied hearts reduced AMPK activity due to the inactivation of its upstream kinase LKB1 by HNE [95,117]. Notably, resveratrol rescued this anti-hypertrophic signalling pathway by blocking the effects of HNE on LKB1 and AMPK in isolated cardiomyocytes as well as SHRs and angiotensin-II infused mice [95,117]. Consistent with the essential role of LKB1 in the regulation of growth and AMPK activity in the heart, cardiac-restricted deletion of LKB1 leads to cardiac hypertrophy in mice [149]. Taken together, this data suggests that the anti-hypertrophic effects of resveratrol on the cardiomyocyte can be regulated by LKB1-AMPK signalling and downstream targets, such as p70S6K and eEF2.

In addition to protein synthesis, the calcineurin-NFAT signaling pathway is also activated in LVH. In response to hypertrophic stimuli, calcineurin dephosphorylates NFAT and promotes its translocation to the nucleus of the cardiomyocyte where it mediates the transcription of numerous targets involved in LVH [150]. Calcineurin-NFAT signalling is activated in a sustained manner during aortic-constriction-induced pressure overload and myocardial infarction-induced heart failure [143]. Cardiac-restricted activation of calcineurin or its downstream effector, NFAT, is sufficient to induce robust LVH in transgenic mice [151]. Notably, resveratrol inhibits NFAT mediated transcription in isolated cardiomyocytes at a dose that prevents increased cell size and protein synthesis [148]. In addition, long-term AMPK activation with AICAR reduced calcineurin-NFAT signalling in rats subjected to transaortic constriction [152]. While numerous pathways could be responsible for these anti-hypertrophic effects, it was demonstrated that resveratrol could not inhibit most of the NFAT-dependent transcription in MEFs lacking AMPK, providing evidence that suppression of the calcineurin-NFAT pathways requires AMPK activation [148].

## 8. Resveratrol and Exercise in Ischemia-Reperfusion Injury

Preconditioning involves the exposure to mild stress (e.g., short episodes of ischemia) that increase the ability of the heart to adapt to subsequent severe stresses such as a longer period of ischemia [153]. Regular physical activity not only prevents ischemic episodes [154,155], but is also believed to precondition the heart to ischemic insults and as a result reduce infarct size and improve post-ischemic functional recovery [156,157,158]. This is thought to involve the activation of survival kinase pathways [159,160] as well as nitric oxide synthesis [161] and oxidative stress resistance [162]. The efficacy of resveratrol as a pharmacologic preconditioning agent has also been demonstrated in several studies involving rats subjected to left coronary artery ligation [163,164,165,166,167,168]. Collectively, this research showed that resveratrol reduced infarct size and improved myocardial function. In addition, resveratrol was able to prevent cardiac arrhythmias and the progression of cardiac hypertrophy induced by myocardial infarction [165,168]. Resveratrol has also been shown to precondition the heart through the activation of survival kinase pathways, which includes post-ischemic activation of Akt [76]. As oxidative stress contributes to ischemic injury, resveratrol also protects the heart from the post-ischemic production of oxidative stress through the reduction of superoxide production [169], lipid peroxides [170,171,172] and the activation of antioxidant defences [76,172]. The production of cardiac NO is likely also involved since L-NAME blocked the ability of resveratrol to improve post-ischemic recovery [167,168]. Overall these findings suggest that resveratrol is of significant cardioprotective benefit in the setting of post-ischemic recovery.

## 9. Resveratrol and Exercise in Heart Failure

Various heart diseases, including myocardial infarction, hypertension, and idiopathic dilated cardiomyopathy contribute to the development of heart failure [173]. Both an active lifestyle and exercise training has been shown to be effective in preventing the development of heart failure in individuals with cardiovascular disease [174]. More recently, systematic reviews of exercise-based interventions for heart failure have concluded that aerobic exercise training improved exercise capacity and reversed ventricular remodeling in clinically stable heart failure patients [174,175,176]. Despite the recommendation of modest physical activity as a component of the management of heart failure patients, the mechanisms underlying the benefits of exercise in heart failure are incompletely understood. Aerobic exercise training augments mitochondrial function, in part by reducing reactive oxygen species levels, increasing NO synthesis and mitochondrial biogenesis (reviewed in [162]). Since the ability of resveratrol to mimic [29] and augment exercise training [22] involves similar adaptations in skeletal muscle and the heart [81,117,133], resveratrol therapy could be as beneficial as exercise for the treatment of heart failure. As a consequence, the effects of resveratrol have been investigated in several different rodent models of chronic heart failure.

In several studies, treatment with resveratrol prior to the onset of cardiac dysfunction prevented the development of heart failure. In the well-characterized Dahl-salt sensitive rat model of heart failure, resveratrol improved survival and prevented a decline in systolic function of the heart [42]. In this model of hypertension-induced heart failure, the effects of resveratrol involved significant improvements in mitochondrial function and fatty acid utilization in the skeletal muscle as well as the heart that involved the activation of PGC1α [42]. In TO-2 hamsters that are genetically predisposed to develop heart failure, resveratrol reduced fibrosis, prevented ventricular dilatation, preserved cardiac function and increased lifespan [177]. Consistent with the key role of oxidative stress in the development of chronic heart failure [178], resveratrol enhanced cardiac SOD2 expression and reduced cardiac reactive oxygen species in the TO-2 hamster heart [177]. Interestingly, accumulation of SIRT1 in the nucleus of the cardiomyocyte was required for the effect of resveratrol in the TO-2 hamster [177]. Resveratrol also attenuated the development of transverse aortic constriction-induced heart failure in rats and reduced lipid peroxidation and fibrosis in the heart [147]. In addition to reducing oxidative stress, resveratrol also reduced macrophage and mast cell infiltration in the hearts of these rats [147]. Consistent with its anti-inflammatory effects, resveratrol also prevented myocardial injury due to myocarditis [179]. Specifically, pre-treatment of rats with resveratrol prevented LVH and preserved cardiac function in myosin-induced autoimmune myocarditis, in association with reduced expression of inflammatory cytokines and increased SIRT1 levels in the heart [179].

In addition to preventing other forms of heart failure, resveratrol has also been shown to prevent doxorubicin-induced heart failure. Doxorubicin is a broad-spectrum anti-tumour chemotherapeutic agent that triggers dose-dependent LV dysfunction that can progress to heart failure [180]. Many reports implicate mitochondrial dysfunction and reactive oxygen species production in triggering cardiomyocyte cell death [181,182]. As predicted, aerobic exercise training prevents LV dysfunction and improves exercise performance in patients undergoing chemotherapy [183,184,185]; effects that likely involve mitochondrial biogenesis and reduced reactive oxygen species [186]. In doxorubicin-treated rats, resveratrol also protects cardiomyocytes from oxidative stress and cell death through improved mitochondrial function, reduced reactive oxygen species, and increased expression of anti-oxidant enzymes [187,188]. As a result, resveratrol prevented doxorubicin-induced fibrosis, ventricular dilation and the decline in systolic function [188]. More recently, the effects of exercise training and resveratrol supplementation were directly compared in female mice with doxorubicin-induced heart failure [35]. Interestingly, treadmill exercise training (45 min/day for 8 weeks) only partially attenuated adverse LV remodeling, while resveratrol (~320 mg/kg/day) completely prevented LV remodeling in doxorubicin treated mice [35]. These findings suggest that while resveratrol and exercise training may offer protection against doxorubicin-induced cardiotoxicity via similar pathways and resveratrol may provide additional benefits through targeting alternative protective pathways in the heart (Table 1). 

Although all of the molecular mechanisms contributing to resveratrol preventing doxorubicin-induced heart failure are likely not known, some pathways have been implicated. For instance, studies have shown that doxorubicin inhibits SIRT1 and AMPK in the hearts of male Wistar rats [189,190] and cultured cells [191], suggesting that the restoration of these two pathways may be of benefit in doxorubicin-induced heart failure. Indeed, administration of resveratrol to doxorubicin-treated cardiomyocytes, increased SOD2 activity through the stimulation of SIRT1 expression [187]. On the other hand, resveratrol did not affect SIRT1 expression in the hearts of doxorubicin-treated female mice, though it increased SOD2 expression and reduced oxidative stress, suggesting that increased SIRT1 expression was not necessary for the effects of resveratrol in this model [35]. Differences between these studies could be attributed to differences in species/models utilized in these different studies, as well as the acute supraclinical dosages of doxorubicin utilized in the preceding studies [189,190,191] compared to the chronic administration of a lower dose of doxorubicin in the female mice [35]. 

In addition to increased SOD2 expression, other pathways also appear to be involved in the protective effects of resveratrol. For instance, while both exercise training and resveratrol attenuated the reduction of electron transport chain complexes I and II in the doxorubicin treated mice, resveratrol also increased the expression of mitofusin-1 and -2, which are required for the formation of a dynamic mitochondrial network in the hearts of doxorubicin treated mice [35]. Together, these findings suggest that optimizing the function of cardiac mitochondria is a central mechanism involved in the benefits of exercise training and resveratrol treatment in mouse models of doxorubicin-induced heart failure.

Although all of the aforementioned studies showing that resveratrol prevented heart failure are important, studies demonstrating that resveratrol can be used to treat heart failure are arguably more clinically relevant. While these latter studies are fewer in number, resveratrol has been shown to reduce infarct size, lessen detrimental cardiac remodeling and improve cardiac function when administered following surgical myocardial infarction [192,193,194,195]. For instance, treatment of rats with resveratrol dramatically increased the survival rate in rats treated following left anterior descending coronary artery ligation-induced myocardial infarction for 16 weeks [192]. This effect appeared to involve the upregulation of SIRT1 and AMPK, as haploinsufficient SIRT1 mice had reduced left ventricular developed pressure and AMPK activity in Langendorff perfused hearts, though this study did not investigate the effect of resveratrol in SIRT1 deficiency and heart failure per se [192]. Resveratrol also reversed remodelling in hearts with large old infarcted regions of the myocardium, when resveratrol was started four weeks following the establishment of heart failure and the resveratrol treatment was administered for two weeks thereafter [195]. In this study, the upregulation of AMPK and cardiac autophagy by resveratrol appeared to be involved since the expression of antioxidant enzymes and the activity of pro-survival kinase pathways were unchanged by resveratrol treatment [195]. Together, these studies show that resveratrol does not only prevent, but may also treat certain types of heart failure.

## 10. Conclusions

Overall, the literature suggests that in a similar manner as exercise, resveratrol provides protection against the development of cardiovascular disorders in rodents. Indeed, resveratrol and exercise training induce positive adaptations on the O_2_ transport system through the stimulation of skeletal and cardiac muscle mitochondrial biogenesis, NO production, oxidative stress resistance and anti-inflammatory properties (Table 1). The biological pathways that mediate these effects are highly interconnected and while we are only beginning to understand the underlying mechanisms, SIRT1, PGC-1α and AMPK appear to be central to the effects of resveratrol on the O_2_ transport system (Figure 1). While resveratrol may mimic certain aspects of exercise, it is still unclear whether or not resveratrol improves exercise performance. Indeed, in some, but not all rodent models, resveratrol improves exercise endurance. On the other hand, human trials do not suggest that resveratrol improves exercise performance. The reason for these discrepancies are currently unclear and more research, particularly in humans, is required to reach clear and unambiguous conclusions about whether resveratrol is beneficial alone or in combination with exercise or whether it is contraindicated in exercise-mediated health benefits. Moreover, our understanding of the interaction of resveratrol with other commonly prescribed pharmaceutical agents is poor. These studies need to be performed before resveratrol may be safely adopted for clinical practice.
